# Editorial for the Special Issue “Biological and Toxicological Studies of Nanoparticles”

**DOI:** 10.3390/nano13131968

**Published:** 2023-06-28

**Authors:** Olivier Joubert

**Affiliations:** Institut Jean Lamour, CNRS 7198, University of Lorraine, 54015 Nancy, France; olivier.joubert@univ-lorraine.fr

Nanoparticles have attracted a great deal of attention over the past two decades or more due to their unique size-dependent physical and chemical properties. They are exploited as promising materials for a wide range of applications, including biological/chemical sensors, food, drugs, bioimaging, optoelectronics, etc.

For the same period, toxicologists have been questioning the impact of this new technology on human health, but also on different biotopes. A very large number of studies have been conducted over the last two decades; however, the data obtained on the toxicity of nanoparticles are contradictory and of variable quality.

In this issue, we aim to present a multidisciplinary overview of recent scientific articles that delve into various aspects of nanotoxicology. Through the exploration of these articles, we seek to highlight the newly developed tools to identify the biological effects of nanoparticles, as well as the antimicrobial, anticancer, or toxic activity of a diverse array of nanoparticles.

Nanoparticles could have antimicrobial effects; Lai et al. identified multiple mechanisms of toxicity induced by copper nanoparticles [1], while Balaz et al. and Salayova et al. developed an alternative method for the green synthesis of silver nanoparticles with antibacterial activities [2,3]. 

To develop new anticancer treatments, Krasteva investigated the biocompatibility/toxicity of PEGylated graphene oxide nanoparticles combined with near-infrared laser irradiation in a colorectal carcinoma in vitro model [4]. 

It must be admitted that the identification of the toxic effects of nanoparticles requires new approaches. From the high-throughput in vitro CometChip Assay [5] to the air–lung interface (ALI) system [6,7], these new techniques broaden the range of tools available to researchers, allowing them to identify the biological/adverse pathways induced after exposure to nano-objects, especially via transcriptomics [8]. In one such ALI model, Hufnagel studied the effects of nanocomposite combustion aerosols [9]. A very recent concept is the adverse outcome pathway (AOP) approach. AOPs provide a systematic way to link mechanistic information with toxicity data, allowing scientists to predict the potential adverse effects of chemicals that have not been extensively studied. AOPs are being actively developed and utilized by regulatory agencies, researchers, and industry stakeholders. Here, Weiss et al. used carbon dots to propose an AOP for acute lung inflammation [10].

Different pathways or effects were identified. Kim SH et al. [11] discovered the skin sensitization potential of silica nanoparticles, Kim IY [8] the ROS production and the apoptosis induced by TiO_2_ in their model, and Valentino the impairment of gene expression in young and elderly rat lungs after nanoTiO_2_ inhalation [12]. Meanwhile, the team of Barthel and Seidel showed that long-term exposure to low concentrations of multiwalled carbon nanotubes could lead to an epithelial mesenchymal transition in BEAS-2B cells [13].

Finally, ecotoxicology was not forgotten, as Kong compared the effect of metal oxide nanoparticles on the growth and roots of two species of plants [14].

To conclude, this Special Issue presents several examples of the latest advancements in assessing nanoparticle toxicity and bioactivity. We hope readers will enjoy reading these articles and find them useful in their research.
